# RETRACTION: Clinical Application of 675 Nm Laser Therapy for Dorsal Hand Skin Hyperpigmentation

**DOI:** 10.1111/srt.70214

**Published:** 2025-07-22

**RETRACTION**: I. Alter, I. Fusco, F. Madeddu, and T. Zingoni, “Clinical Application of 675 nm Laser Therapy for Dorsal Hand Skin Hyperpigmentation,” *Skin Research and Technology* 29, no. 10 (2023): e13484, https://doi.org/10.1111/srt.13484.

The above article, published online on 4 October 2023 in Wiley Online Library (wileyonlinelibrary.com), has been retracted by agreement between *Skin Research and Technology*; and John Wiley & Sons Ltd. The article was accepted for publication following an insufficient peer review process that does not meet the standards of the journal's peer review policy. Furthermore, scientific inconsistencies were found. Finally, the article lacks a statement confirming that the study has been approved by an appropriate institutional review board or ethics committee. As a result, the editorial team cannot vouch for the study's adherence to ethical research standards nor for the reliability of the study's conclusions. Accordingly, the article has been retracted. The authors have been informed of this decision.

